# Annotations

**Published:** 1903-01-10

**Authors:** 


### ANNOTATIONS.

### Co-operative Practice.

THERE is something very striking in the different amount of "plant" required by workers in different parts of the medical field. Some men, with nothing more in the way of tools than a stethescope and perhaps a thermometer, are able to make their way, while others seem to find it necessary to decorate their consulting-rooms with all kinds of fearsome apparatus. No specialist of modern times, however, comes up to the electro-therapeutist in the amount of "plant" which he wants if he is really to put in practice the treatment he prescribes. Among all the London hospitals—institutions in which everything is done on a large scale—there probably are not half a dozen which possess a really complete and up-to-date installation for the electrical treatment of disease. How then can a young and struggling electro-therapeutist afford to surround himself with all the necessary apparatus? For the due conduct of such a speciality he would require the use of an electro-therapeutic institute rather than the modest "consulting-room with use of waiting-room," which, with a battery or two, is all that he can afford. The position of such a practitioner is almost as pathetic as it is absurd, and we can but welcome with all the sympathy it deserves—a somewhat modified sympathy after all—a suggestion made by Mr. Edmund Owen in addressing the British Electro-Therapeutical Society, to the effect that the members of the Society should combine and set up a central co operative establishment, fitted with all the most modern electrical apparatus, for the use of the members in their practices; the whole to be conducted on a business basis in the form of a limited liability company, the shareholders being the members of the Society. From a business point of view we can but say "admirable!" What the Royal College of Physicians would say to such a proposal is, however, another affair. That ancient Corporation has laid down certain definite rules for the conduct of its members, which, if carried out in their spirit as well as to the letter, would go far we fancy towards depriving Mr. Owen's system of co-operative practice of that professional sanction without which, as medical affairs in London are at present organised, would be essential for its permanent success.

### Mankind in the Marring.

THE recently issued report of the proceedings of last summer's International Congress for the Welfare and Protection of Children affords abundant evidence of the keen attention and well-directed effort now concentrated on all matters concerning the well-being of the young life of the country. As Professor Sully has shown in his fascinating "Studies in Childhood," we require a more careful study of the unfolding of mind in the child if so-called education is to be anything more than the baldest empiricism. At the present time manifold influences are hindering physical efficiency and hampering mental development, and this is true to a large extent for all classes of society. There is urgent need for more effectual measures which may adequately protect infant life; but above all it is imperative that the enlightenment of parents and those responsible for the protection and upbringing of the young human subject should be secured. It is lamentable to have to admit that a great part of the present high rate of infant mortality and much of the maiming of body and marring of mind is the direct outcome of the ignorance and apathy of those who stand, both by the ties of nature and the rules of social life, in the place of guardians. But while pity is well, although not always wisely, bestowed upon the offspring of the poor, the burden of the little prisoner in many a wealthy home is only too frequently lost sight of by the thoughtless and unheeding. Mr. Charles Booth in his "Life and Labour of the People" has shown that in opportunities for mental impressions the children of the labouring classes and the offspring of poor parents are usually more advantageously circumstanced than the lonely child of the well-to-do, imprisoned in its secluded nursery, isolated from what should be the educational movements of the home and shut away from the mind unfolding environment of a bright and quickly-changing world of action. Mr. H. G. Wells, writing in *The Cosmopolitan* makes a very much-needed protest against the mentally stultifying and hampering influence of the foreign nurse or nursery governess in the home of the wealthy. Such a servant instead of helping the young life to a rapid and correct appreciation and use of its mother tongue does much to encourage and develop barbarism of speech, and inevitably leads to a lamentable restriction, and it may be more or less permanent limitation in mental progress.

### Tetanus Infection in Gelatine.

THE injection of gelatine as a homostatic agent has occasionally been followed by the occurrence of tetanus, and it has been somewhat of a puzzle to ascertain why this unfortunate accident should have taken place. Gelatine is made commercially from bone, cartilage, tendons, ligaments, and clippings of hide, and as all these are boiled for a long time in water, it is clear that all infection resident in them must be destroyed by the heat to which they are subjected in this part of the process. When, however, the solution of gelatine is drawn off and allowed to cool it "sets" into a jelly, and this, after being cut into strips or sheets, is placed on wire netting to dry, and it is in this stage that it runs risks of infection. As is well known, the home of the tetanus bacillus is the earth, the microbe being more especially associated with the stable and with manured fields, and as the manufacture of gelatine usually takes place in connection with proceedings for the utilisation of various products of the slaughter-house and the knacker's yard, it is not difficult to understand that during the time the jelly is exposed to the air with the object of being dried, it is also exposed to the dust arising from contaminated soil, and thus may in some cases become infected with the bacillus of tetanus. An examination of seven samples of commercial gelatine lately undertaken in the United States Government laboratory resulted in one of these samples showing tetanus spores, while in two others bacilli were found which it would be hard to distinguish from those of tetanus although their identity was not definitely proved. Clearly, then, all gelatine intended for injections should be fully sterilised in the laboratory either by prolonged boiling, or by the fractional method by repeated heatings on three successive days.

